# Trial of a Novel Oral Cannabinoid Formulation in Patients with Hypertension: A Double-Blind, Placebo-Controlled Pharmacogenetic Study

**DOI:** 10.3390/ph16050645

**Published:** 2023-04-25

**Authors:** Ana Batinic, Davorka Sutlović, Sendi Kuret, Antonela Matana, Marko Kumric, Josko Bozic, Zeljko Dujic

**Affiliations:** 1 Pharmacy of Split-Dalmatia County, 21000 Split, Croatia; 2 University Department of Health Studies, University of Split, 21000 Split, Croatia; 3 Department of Toxicology and Pharmacogenetics, School of Medicine, University of Split, 21000 Split, Croatia; 4 Department of Pathophysiology, University of Split School of Medicine, 21000 Split, Croatia; 5 Department of Integrative Physiology, School of Medicine, University of Split, 21000 Split, Croatia

**Keywords:** cannabidiol, blood pressure, CYP P450 genes, GC-MS analysis, SNP genotyping

## Abstract

Cannabidiol (CBD) is a non-psychoactive cannabinoid, and available evidence suggests potential efficacy in the treatment of many disorders. DehydraTECH™2.0 CBD is a patented capsule formulation that improves the bioabsorption of CBD. We sought to compare the effects of CBD and DehydraTECH™2.0 CBD based on polymorphisms in CYP P450 genes and investigate the effects of a single CBD dose on blood pressure. In a randomized and double-blinded order, 12 females and 12 males with reported hypertension were given either placebo capsules or DehydraTECH™2.0 CBD (300 mg of CBD, each). Blood pressure and heart rate were measured during 3 h, and blood and urine samples were collected. In the first 20 min following the dose, there was a greater reduction in diastolic blood pressure (*p* = 0.025) and mean arterial pressure MAP (*p* = 0.056) with DehydraTECH™2.0 CBD, which was probably due to its greater CBD bioavailability. In the CYP2C9*2*3 enzyme, subjects with the poor metabolizer (PM) phenotype had higher plasma CBD concentrations. Both CYP2C19*2 (*p* = 0.037) and CYP2C19*17 (*p* = 0.022) were negatively associated with urinary CBD levels (beta = −0.489 for CYP2C19*2 and beta = −0.494 for CYP2C19*17). Further research is required to establish the impact of CYP P450 enzymes and the identification of metabolizer phenotype for the optimization of CBD formulations.

## 1. Introduction

Cannabidiol (CBD) is a bioactive cannabinoid of the plant *Cannabis sativa* L. Unlike tetrahydrocannabinol (THC; Δ^9^-tetrahidrocannabinol), CBD has non-psychotropic effects and is consumed as a food supplement by millions of people today [1,2]. There are over 900 studies, reported on ClinicalTrials.gov, exploring the potential indications of CBD on Parkinson’s disease, stroke, inflammations, epilepsy, chronic pain and many psychiatric conditions. CBD has a favorable low-affinity safety profile for CB1 and CB2 receptors that represent the primary binding site of THC (the primary psychoactive ingredient of cannabis) [3,4]. Available evidence suggests the effectiveness of CBD in various diseases including epileptic seizures (especially pediatric patients), inflammatory conditions, malignancies, chronic pain, schizophrenia, psychosis, and cardiovascular disease [5,6,7,8,9,10,11,12,13,14]. Stressful situations are associated with increased blood pressure and heart rate. CBD can alleviate both, but it can also improve vascular endothelial function and decrease arterial stiffness [15]. Good tolerability of high doses (1500 mg to 6000 mg) of purified oral CBD preparations (100 mg/mL; Epidiolex^®^, GW Pharmaceuticals, Cambridge, UK: pure CBD oral solution) without serious side effects (only with occasional mild to moderate side effects such as nausea, diarrhea, headache and somnolence) has been demonstrated, although these are doses that are more than ten times higher than the standard recommended doses [16]. Safety has also been demonstrated at 20 mg/kg daily for 2–3 months [6]. The doses used in more recent studies are drastically lower and amount to about 5% of the maximum applied doses in tolerability trials. Healthy adults consuming CBD may experience elevated liver enzymes (ALT), which is not negligible today when more and more CBD products are available over-the-counter on the market [17]. Novel formulations for improving oral CBD delivery and efficacy are under development in the pharmaceutical industry [18]. Some preparations have technologically advanced formulations that improve bioavailability and allow higher concentrations of CBD in plasma. For instance, faster and higher absorption of CBD was shown in young volunteers after oral TurboCBD™ formulation, which led to a decrease in diastolic and mean arterial pressure and increase in cerebral perfusion [19]. In this study, we used DehydraTECH™2.0 CBD, a patented capsule that advances the earlier TurboCBD™ formulation (developed by Lexaria Bioscience Corp., Kelowna, BC, Canada) and increases the bioabsorption of the active content due to its enhanced lipophilic composition by bypassing (or reducing) first-pass liver metabolism [19,20,21]. The bioavailability of CBD in subjects depends also on the rate of metabolism associated with the genetic variability of cytochrome P450: CYP2C9*2, CYP2C9*3, CYP2C19*2, CYP2C19*3, CYP2C19*7 and CYP3A4 genes [22,23,24,25,26,27,28]. The main objective of this randomized, placebo-controlled crossover study was to investigate the effects of a single CBD dose on blood pressure in patients with untreated arterial hypertension and to compare the effect of generic CBD and DehydraTECH™2.0 CBD formulation on polymorphisms in CYP P450 genes. The hypothesis is that the hypotensive effects of CBD will be more apparent in advanced formulation DehydraTECH™2.0 CBD comparing to the generic CBD control tablets and that 300 mg of CBD is a safe dietary supplement for stage 1 or stage 2 hypertensive population.

## 2. Results

### 2.1. Visual Analog Scale (VAS)

A total of 24 subjects, 12 female and 12 male, participated in this study, and they were included for statistical analysis of the VAS data. The results are presented in Table 1.

VAS was performed on a scale from 0 to 100—i.e., 0 being no distress and 100 being unbearable distress. While there does not appear to be any differences between the doses on VASs, there was a tendency for anxiety to decrease (main effect *p* = 0.083) and sleepiness to increase (main effect *p* < 0.001) with both doses across the 3 h. Statistical significance was assessed using a linear mixed model, with Bonferroni adjusted post hoc tests.

### 2.2. CBD Concentrations

The results of concentration CBD and DehydraTECH™2.0 CBD were statistically analyzed by measurement range (minimum and maximum values, median and interquartile range (IQR)) for every group of samples. The results are presented in Table 2.

**Figure 1:**
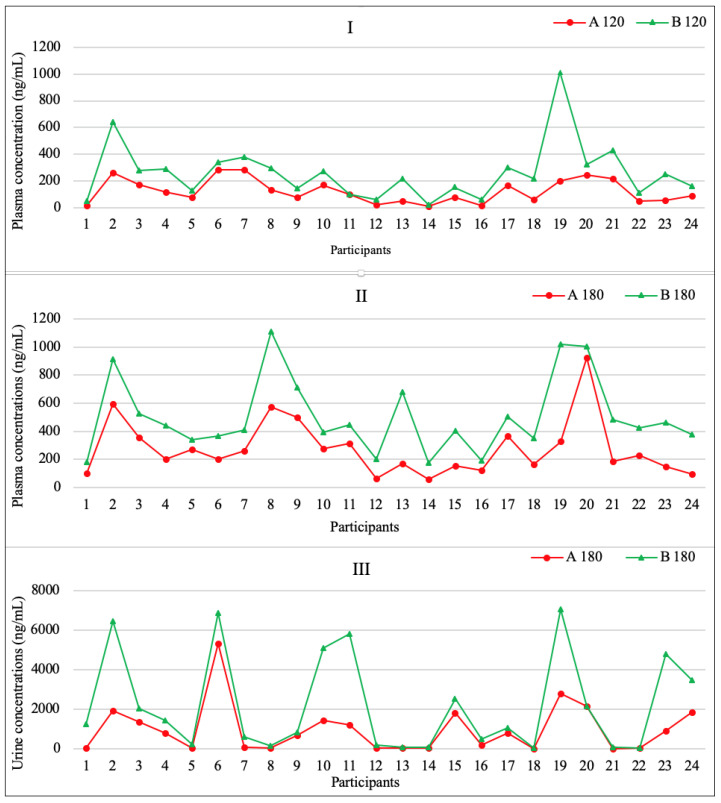
Plasma cannabidiol (CBD (dose A) and DehydraTECH™2.0 CBD (dose B)) concentration in venous blood (**I**,**II**) and urine (**III**). Individual data for 24 participants. Concentrations in plasma samples taken in 120th minute (**I**) and in 180th minute (**II**) after ingestion and in urine samples taken in 180th minute (**III**) after ingestion.

No statistical difference was found between the test participant’ CBD concentrations for formulations A and B for any of the tested samples using the Mann–Whitney test. The investigated samples had *p* values greater than 0.05: for plasma samples at 120 min. (*p* = 0.853), for plasma samples at 180 min. (*p* = 0.322), and for urine samples at 180 min. (*p* = 0.680). In terms of sex, there was no statistically significant difference in the CBD concentrations in plasma and urine (formulation A). After the consumption of formulation B, no statistically significant difference was observed in plasma CBD concentrations, while the concentration of CBD in urine at 180 min, with respect to gender, showed a statistically significant difference with higher CBD concentration observed in men (*p* = 0.021). Figure 1 shows a comparison of plasma cannabidiol: CBD (dose A) and DehydraTECH™2.0 CBD (dose B) concentration in venous blood samples (**I** and **II**) and urine samples (**III**).

### 2.3. The Effect of CBD on Blood Pressure (BP)

**Figure 2:**
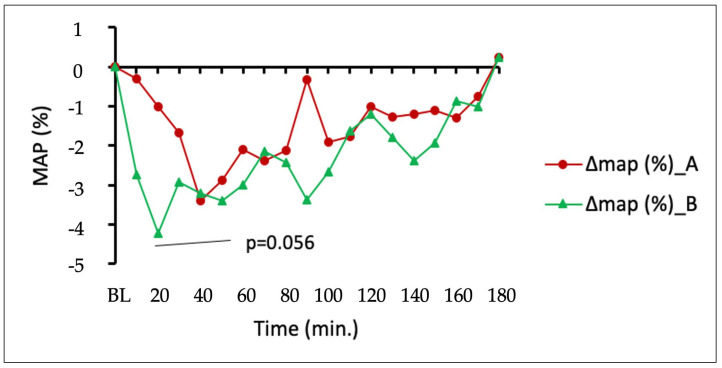
Changes in mean arterial blood pressure (MAP) between generic CBD control (dose A) and DehydraTECH™2.0 CBD (dose B). Data are grouped means (n = 24) with linear regression.

The blood pressure and heart rate of all subjects after consuming different CBD formulations were measured every 10 min. Figure 2 shows a comparison of average changes in MAP (mean arterial pressure) in the measured time.

There was a tendency for relative mean arterial pressure (MAP) to be reduced to a greater extent from baseline with the DehydraTECH™2.0 CBD than the concentration matched, generic CBD control, most notably in the initial 20 min post-dosing.

**Figure 3:**
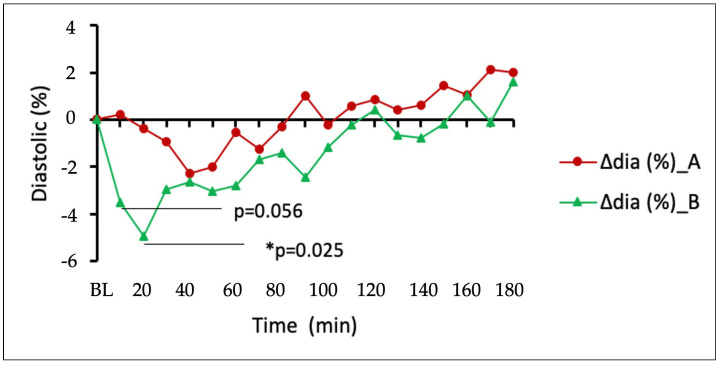
Changes in diastolic blood pressure between generic CBD control (dose A) and DehydraTECH™2.0 CBD (dose B). Data are grouped means (n = 24) with linear regression.

Regardless of the fact that the plasma concentration was not measured 20 min after consumption, and it is unknown whether the maximum concentration was reached at that time, a DehydraTECH™2.0 CBD had greater influence on relative diastolic blood pressure drop than the generic CBD control (Figure 3).

**Figure 4:**
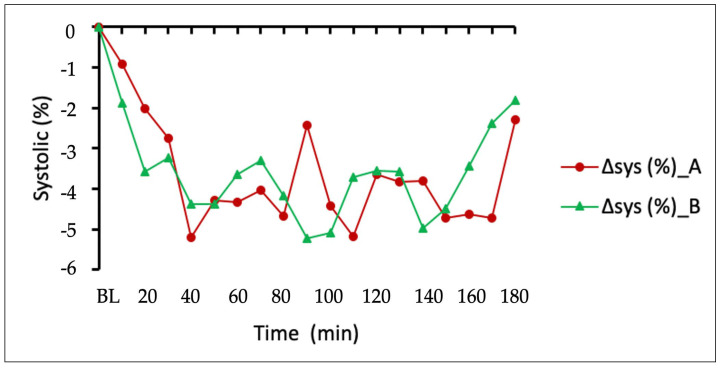
Systolic BP remained depressed throughout the entire 3 h duration of the study for both formulations.

This was most notable in the initial 10–20 min period post-dosing evidencing statistical significance at the 20 min timepoint (*p* = 0.025). Both formulations reduced systolic pressure throughout the whole measuring period (Figure 4), and there was no statistically significant difference in their effects on systolic pressure (*p* > 0.05).

Heart rate reduction was statistically different between the two studied at 180 min (CBD, *p* = 0.024; DehydraTECH™2.0 CBD, *p* = 0.020); however, at 120 min, only the DehydraTECH™2.0 CBD formulation was statistically different (*p* = 0.048) (Table 3). Compared to the baseline, no statistically significant difference was observed in changes in systolic and diastolic blood pressure at 120 and 180 min from the start for any of the tested formulations.

### 2.4. SNP Genotyping

The results of genotyping of six investigated SNP loci in DNA samples of participants are given in Table 4. According to the Hardy–Weinberg equilibrium, there was no statistically significant difference for any of the studied SNPs (*p* value was in range from 0.243 to 0.959). The results for the minor allele frequency (MAF) are consistent with the data presented in the PharmGKB resource [29].

### 2.5. Association of CBD Concentrations with CYP Genotype and Phenotype of Subjects

Since none of the concentration values from formulation A and B plasma samples taken at 120 and 180 min from the start and formulation A and B urine samples taken at 180 min were normally transformed, a logarithmic transformation of the data was performed in order to produce a normal distribution. A linear regression model was performed for each of the samples log (formulation A and B plasma samples taken at 120 and 180 min) and log (formulation A and B urine samples taken at 180 min) as dependent variables, and the independent variables were CYP2C9*2, CYP2C9*3, CYP2C19*2 and CYP2C19*17.

For formulation A, no model (for log values of plasma samples at 120 and 180 min and urine samples at 180 min) was statistically significant: *p* values were, respectively, *p* = 0.821, *p* = 0.738 and *p* = 0.196.

For formulation B, the linear regression model (for log values of formulation B urine samples at 180 min) was also adjusted for gender.

Without distribution by phenotype, the linear regression model was not significant for log values of formulation B plasma samples at 120 min (*p* = 0.863) and the log values of formulation B plasma samples at 180 min (*p* = 0.979). For the formulation B log values of urine samples at 180 min, the model was statistically significant (*p* = 0.028, R2 = 0.3332). The variants CYP2C19*2 (*p* = 0.037) and CYP2C19*17 (*p* = 0.022) showed statistical significance. Both were negatively associated with urinary CBD levels (beta = −0.489 for CYP2C19*2 and beta = −0.494 for CYP2C19*17). Each mutated allele reduces the level of CBD.

**Figure 5:**
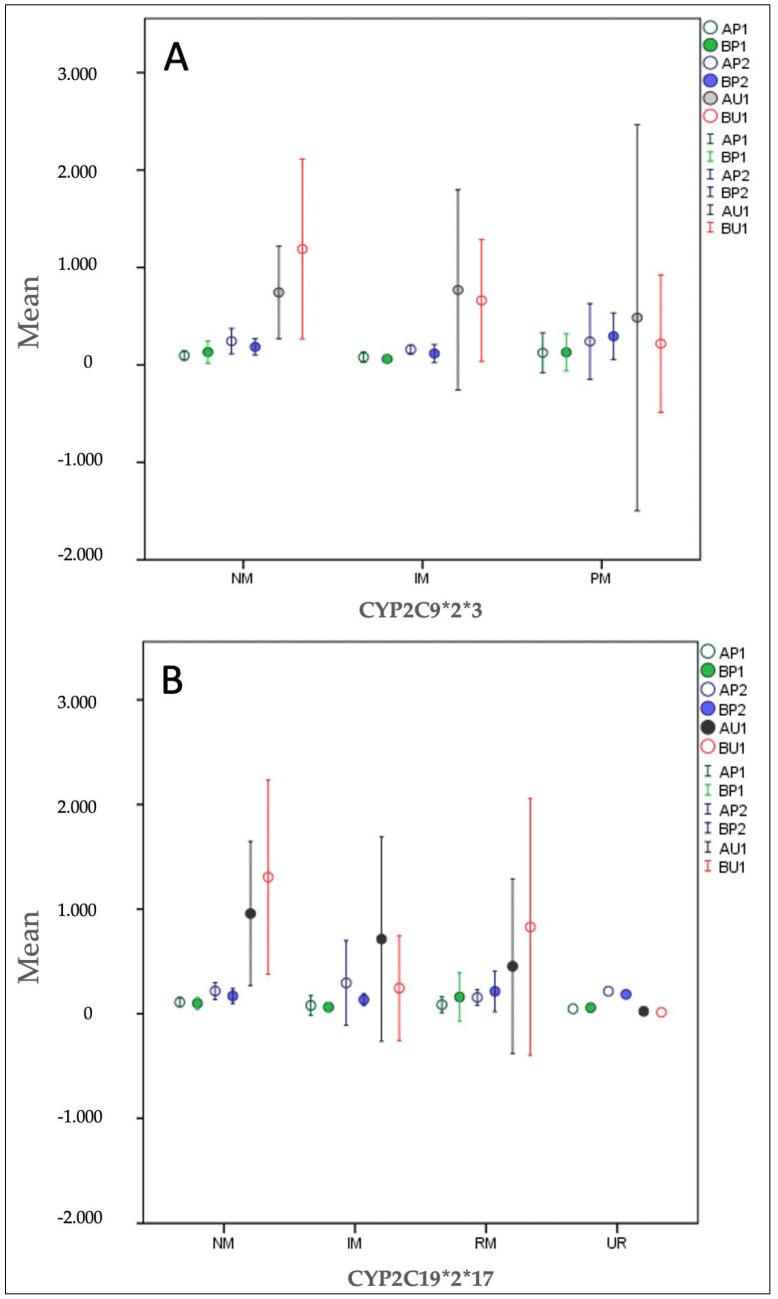
Mean values of CBD plasma (AP1 and BP1 at 120 min; AP2 and BP2 at 180 min) and urine (AU1 and BU1 at 180 min) concentrations (ng/mL) after consumption of formulation A and B classified by CYP2C9*2*3 phenotype: normal metabolism (NM), intermediate metabolism (IM) and poor metabolism (PM)) (**A**) and CYP2C19*2*17 phenotype: normal metabolism (NM), intermediate metabolism (IM), rapid metabolism (RM) and ultra-rapid metabolism (UR), (**B**) (n = 24).

By combining the CYP2C9*2 and CYP2C9*3 as well as CYP2C19*2 CYP2C19*17 genotypes, phenotypes were created for each subject, and thus, phenotypes are also related to drug metabolism (Figure 5) [25,30].

According to the CYP2C9*2*3 enzyme phenotype, there were 13 (54%) subjects with normal metabolism (NM) (genotype *1/*1), eight (33%) with intermediate metabolism (IM) (genotype *1/*2 or *1*3) and 3 subjects (13%) with poor metabolism (PM) (genotype *2/*2, *2/*3 or *3/*3). According to the CYP2C19*2*17 enzyme phenotype, there were 11 (46%) subjects with normal metabolism (NM) (genotype *1/*1), 5 (21%) with intermediate metabolism (IM) (genotype *1/*2 or *2/*17), 7 subjects (29%) with rapid metabolism (RM) (genotype *1/*17) and 1 subject (4%) with ultra-rapid metabolism (UR) (*17/*17).

The variants CYP2C19*2 and CYP2C19*17 showed statistical significance, considering the concentrations of CBD. Therefore, the relationship between changes in blood pressure and their variants has been explored. It has been noted that there is a statistically significant decrease (*p* = 0.033), comparing with baseline, for diastolic pressure for CYP2C19*2. Regarding the genotype of the individuals, there was no statistically significant correlation between CBD concentrations and changes in blood pressure and heart rate for CYP2C19*17.

## 3. Discussion

### 3.1. CBD Effect on Cardiovascular System

This double-blinded, placebo-controlled study examined the impact of acute dosing of a concentration-matched CBD and DehydraTECH™2.0 CBD formulation in male and female subjects with stage 1 or 2 arterial hypertension. Our results showed that after consuming the tested formulations, there was no statistically significant difference in the changes between systolic and diastolic blood pressure at 120 and 180 min. Additionally, DehydraTECH™2.0 CBD tended to lower relative diastolic pressure from baseline more than the concentration-matched, generic CBD control. This was particularly noticeable in the first 10 to 20 min after dosage, with statistical significance being shown at the 20 min timepoint. Additionally, the DehydraTECH™2.0 CBD had a propensity to also reduce relative mean arterial pressure (MAP) from baseline more than the concentration-matched, generic CBD control, particularly in the first 20 min after dosage (*p* = 0.056). It should be noted that the concentration was not measured at the 20th minute, and it is unknown whether the DehydraTECH™2.0 CBD formulation had maximum plasma concentrations at that time. However, the effect on diastolic blood pressure and MAP lowering was most evident at that time. Because of the improved properties of DehydraTECH™2.0 CBD, bioabsorption and bioavailability are increased [19,20,21]. Perhaps it is reasonable to infer that the maximum concentration, and hence the effect, is reached sooner.

A statistically significant decrease was observed for heart rate (HR) for both formulations. At 120 and 180 min, the DehydraTECH™2.0 CBD formulation showed a statistically significant drop in HR, whereas concentration-matched CBD only showed it at 180 min. By comparison, in the previous human clinical study, 120 min were required to achieve the same level of MAP reduction, demonstrating the superior rapidity of onset of the DehydraTECH™2.0 CBD formulation used in the present study, relatively speaking [19].

As the DehydraTECH™2.0 CBD formulation is improved and bypasses the metabolism of the first pass through the liver, it very likely affected the speed of effect of such a formulation. Depending on the formulation, the time of maximum concentration and half-life might differ significantly [1]. For example, Abbotts et al. [1] examined the pharmacokinetics of six different formulations of CBD in 14 subjects and found that the median Tmax was between 30 and 90 min, and that of t1/2 was between 106 and 246 min. In contrast to the timepoint of the greatest BP-lowering effect of CBD, plasma concentrations were higher at later timepoints, e.g., in 180 min samples. Therefore, we assume that the maximum effect of CBD on lowering blood pressure is not related to the moment of maximum plasma concentrations, which we suspect we failed to detect and measure for the DehydraTECH™2.0 CBD formulation in this study if it likely occurred earlier than the 120 and 180 min timepoints of our plasma analyses. Bonomo et al. in their study of nine subjects determined that the concentration of all tested analytes increased up to 2 h after administration [31]. They observed significant inter- and intra-individual variability. The results of our study showed that maximum concentrations were reached after 2 h (120 min). In addition, there was a distinction between the respondents in our findings. While the interindividual differences in the 180th minute were less significant, subjects 2 and 19 had considerably higher levels of CBD in their plasma at the 120th minute than the other participants. These results will undoubtedly need to be further investigated in order to improve CBD supplement dosage and produce a longer-lasting effect without requiring the enhancement of single dosages.

Sultan et al. (2020.) examined the effects of CBD on 26 men and found that the BP-lowering effect of CBD was lost after repeated dosing because tolerance probably developed while endothelial function improved [15]. We assume that this is the reason why in our study, the diastolic pressure values at 180 min were higher than the values at the baseline point, although the CBD concentrations of both formulations were higher at the 180th minute than at the baseline and at the 120th minute [15].

The dose of 300 mg of CBD was chosen based on the results of previous studies [1,5,15,19,32]. The achieved concentrations are shown in Table 2. Although no statistically significant difference was observed in the concentrations of both tested formulations, it is worth noting that the concentration of CBD in plasma of both formulations increased with time. At 120 min, the average concentrations of DehydraTECH™2.0 CBD formulations were higher, while at 180 min, they were lower than those of the concentration-matched CBD. In addition, the new formulation had a larger average urine concentration at 180 min, which may be due to its increased bioresorption [33]. However, with the DehydraTECH™2.0 CBD formulation, almost every subject had a higher plasma CBD concentration (at 120 min and plasma and urine samples at 180 min). This effect seems to be due to the DehydraTECH™2.0 CBD formulation having a higher bioavailability than concentration-matched CBD. Only the urine samples showed a statistically significant difference between the CBD concentrations of the DehydraTECH™2.0 CBD formulation when the gender was taken into consideration. Men specifically had larger CBD concentrations than the female participants (in plasma samples at 120 and 180 min as well as in urine samples at 180 min). It is interesting that Sultan et al. excluded female participants from their study in order to rule out the possibility that gender differences could affect CBD’s effect [15]. However, at the same time, this was their study limitation, and our study provides experimental evidence in sex differences in CBD metabolism.

The heart rate was the primary indicator of elevated CBD concentrations, which steadily dropped, with a remark that the results following intake of the DehydraTECH™2.0 CBD formulation were marginally greater than the CBD concentration-matched formulation. Furthermore, it was observed that the DehydraTECH™2.0 CBD formulation had a better effect on the initial reduction in diastolic blood pressure, MAP and heart rate than the CBD concentration-matched formulation.

### 3.2. SNP Genotyping

Multiallelic genetic polymorphisms depend on ethnicity and are often associated with variations in drug response among populations [23,24,26,34], which in their combinations lead to different pharmacogenetic phenotypes. The results of the polymorphisms of the subjects from this study are consistent with the results found in the PHARMGKB resource [29]. The phenotype of the study subjects, as measured by their MAF frequencies, is consistent with the Caucasian–European population. This is because the CYP2C9*2*3 and CYP2C19*2*17 gene polymorphisms are combinations which result in different phenotypes.

Our results showed that there was no association of CBD concentrations of concentration-matched CBD formulation with any phenotype, while for the DehydraTECH™2.0 CBD formulation, a statistical association was observed between the concentrations of plasma samples extracted at 180 min and the CYP2C9*2*3 phenotype. The IM phenotype had the lowest level, followed by NM, and the PM phenotype had the highest level.

In order to observe the connection between concentrations and metabolism with the genetic variability of cytochrome P450, the research included the analysis of polymorphism CYP2C9*2, CYP2C9*3, CYP2C19*2, CYP2C19*3, CYP2C19*17 and CYP3A4 genes [22,35].

Although numerous studies have suggested an association of genetic variability with CBD metabolism, our results did not fully confirm these findings [17,35]. Our results showed that there was no significant difference in CBD concentrations with genotypes of all SNPs tested individually, unlike combinations.

The results obtained for CYP3A4 and CYP2C19*3 genes were not statistically significant. Beers et al. conducted an in vitro study in which they examined the influence of CYP-450 enzymes on CBD metabolism [35]. Their results showed that both CYP2C9 and CYP2C19 are important participants in the metabolism of CBD to the active metabolite 7-OH-CBD, while CYP3A4 was not involved in the formation of 7-OH-CBD. In our study, the highest association was observed for CYP2C9*2 and CYP2C19*17. In the CYP2C9*2*3 enzyme, subjects with the poor metabolizer (PM) phenotype, compared to other phenotypes, after consuming the DehydraTECH™2.0 CBD formulation had a higher plasma concentration of CBD in 180 min. As a result, consideration for the subject’s phenotype should be given while determining the dosage of DehydraTECH™2.0 CBD formulation.

### 3.3. Medication Influence

Out of all 24 participants, 18 of them were not receiving any therapy. The following medications were being taken by the six subjects: two subjects were taking 25 μg of levothyroxine daily, one subject regularly was taking 100 μg of levothyroxine along with 1 mg of lorazepam, one subject was using 5 mg of diazepam, one subject was using 100 mg of celecoxib, and one subject regularly was taking 100 mg of acetylsalicylic acid per day. According to the results of previous research and available data, the oxidative metabolism of diazepam and lorazepam is mediated by CYP3A4 and CYP2C19 isoenzymes [36]. Celecoxib is primarily metabolized by CYP2C9 [37]. Levothyroxine reduces the activity and expression of CYP3A4 and may influence the pharmacokinetics of concomitant CYP3A substrate drugs [38].

By comparing the concentrations of CBD of the tested formulations in the plasma and urine samples of the mentioned subjects with the mean values from Table 5, it was observed that the values mostly deviate from the mean values. We conclude that the use of these drugs affects the metabolism of CBD, which needs to be taken into consideration of optimizing the dosage of CBD as a dietary supplement in different formulations.

### 3.4. Side Effects

DehydraTECH™2.0 CBD and the concentration-matched, generic CBD control were well tolerated by all subjects, with no serious side effects observed or reported. Ingestion of the concentration-matched, generic CBD control, on the other hand, resulted in mild side effects in some of the volunteers, namely gastrointestinal distress including diarrhea (n = 2). Some volunteers reported relaxation and sleepiness (n = 2) after generic CBD control, while relaxation without sleepiness was reported after DehydraTECH™2.0 CBD (n = 2).

### 3.5. Study Limitations

Limitations of the study include the relatively small number of subjects, acute intervention, no placebo control and no determination of CBD metabolite concentrations. Additionally, the limiting factor for the interpretation of the results is that the concentration in the plasma was not determined more frequently after consumption. Therefore, our follow-up study has now included a larger sample size, placebo control, longer intervention (5 weeks) and the determination of the concentration of the main metabolites of CBD [21].

## 4. Materials and Methods

### 4.1. Participants

Twenty-seven untreated participants with reported or measured stage 1 or stage 2 hypertension (13 female and 14 male) were recruited, and 24 completed all experimental parts.

### 4.2. Anthropometrics and Background

One participant (1 female) dropped out because of syncope, and two participants (2 males) dropped out because their BP was too low (under normal values of 120/80 mm Hg) at the start of the study. Exclusion criteria included: body mass index under 35 kg/m^2^ (according to the National Institute of Health: BMI of 18.5–24.9 kg/m^2^ is considered normal weight; BMI of 25–29.9 kg/m^2^ is considered overweight; BMI of >30 kg/m^2^ is considered obesity), secondary forms of hypertension, if they had any previous history of kidney, gastrointestinal (GI), liver, cardiopulmonary or cerebrovascular disease, epilepsy, diabetes, pregnant or breastfeeding, clinically diagnosed anxiety or depression or if they were taking estrogen supplementation or other prescription drugs or over-the-counter supplements. Participants were also excluded if they were current smokers or had any history of smoking, opioid use or using vapor-based products and medical or recreational use of cannabis. Trained athletes were also excluded. Inclusion criteria included: normal or overweight (body mass index between 18 and 35 kg/m^2^), between the ages of 45 and 70; under 150 min of moderate-to-vigorous activity per week, reported or measured elevated blood pressure (120 to 129 mm Hg systolic and less than 80 mm Hg diastolic), stage 1 hypertension (130/80 to 139/89 mmHg) or stage 2 hypertension (140/90 to 159/99 mmHg) [39]. Participant background information are presented in Table 6.

**Figure 6:**
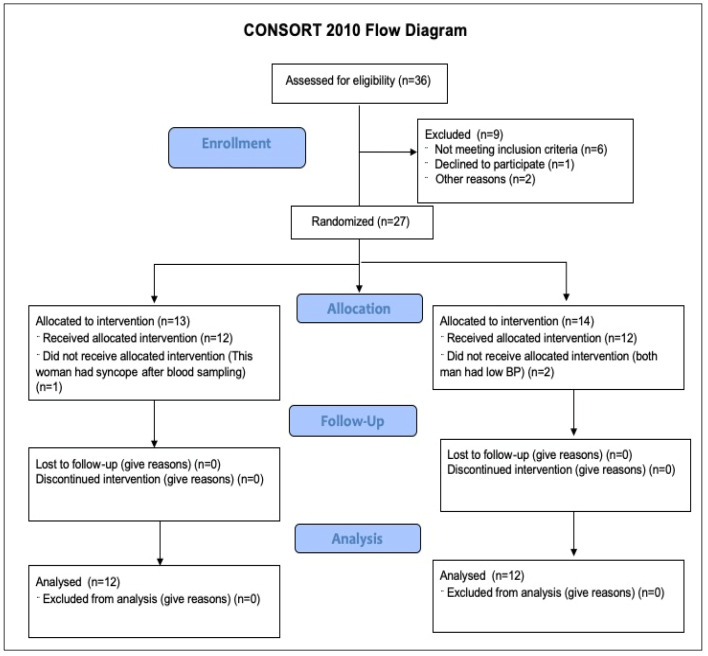
CONSORT flow diagram.

The study design, reporting and implementation followed the CONSORT guidelines and a flow diagram (Figure 6).

Both researchers and subjects were blinded to the performed conditions on testing days by having a research associate who dispatched the capsules to participants according to the randomization schedule and was not involved in any other aspect of the study. When reporting to the laboratory in the morning, participants were in a fasted state (no food or drinks for at least 10 h).

### 4.3. Research Design

This was a double-blinded (Participant, Investigator), cross-over study in which 24 eligible volunteers (12 males and 12 females) visited the laboratory on three occasions.

On the first initial visit (screening), potential participants read through the information and consent form, and they provided written informed consent before any measurements. Following informed consent, the Medical Screening Questionnaire was administered to confirm eligibility criteria. Anthropometric and physiologic measurements were collected (height, body weight, waist circumferences, blood pressure) for baseline participant characterization. All subjects were instructed to keep a food and physical activity diary in the 24 h preceding their second laboratory visit and to replicate food consumption and physical activity in the 24 h preceding the last visit. Subjects were instructed to refrain from caffeine and alcohol-containing drinks for 24 h before each laboratory visit, and dietary logs were collected and corroborated. Eligible participants were then randomized. The sequence of conditions the participant received was generated by a research randomizer web-service (https://www.randomizer.org, accessed on 16 April 2021).

**Figure 7:**
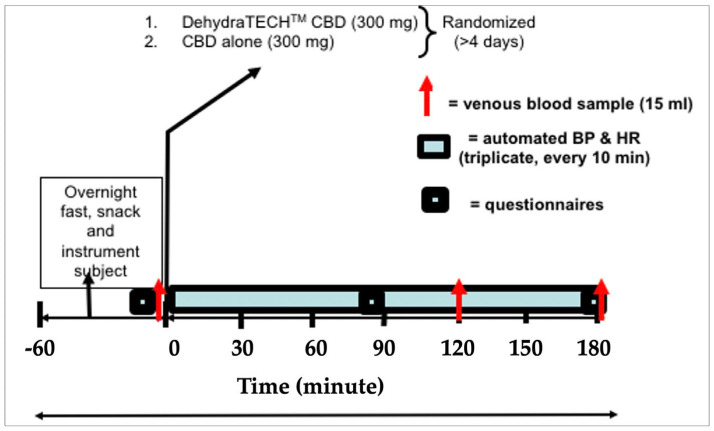
Schematic for Study. The full planned protocol lasted <4 h. Participants arrived in the laboratory after an overnight fast. A snack high in fat (muffin) was provided upon initial arrival. A 15 mL blood sample (red arrows) was collected at baseline and following 120 and 180 min. Automated blood pressure and heart rate were obtained, in triplicate, every 10 min. Before dosing and every 90 min thereafter, standardized questionnaires were used to assess GI symptoms and anxiety. See text below for dosing details.

Second and third visits were experimental trials, each separated by at least 4 days. There were three venous blood samples (15 mL each) collected; one sample was obtained at baseline and the others were obtained at 120 and 180 min following ingestion of the capsules (Figure 7). Blood was drawn into lithium–heparin and EDTA tubes for the measurement of plasma samples before being frozen in a −20 °C research refrigerator. Blood pressure and heart rate were measured in triplicate every 10 min throughout the study. Before dosing and every 90 min thereafter, standardized questionnaires were used to assess GI (gastrointestinal) symptoms and anxiety (visual analog scale—VAS).

The second visit was scheduled at least 24 h after the first visit to the laboratory, and participants were required to be in the laboratory for at least 3.5 h. Participants had to report to the laboratory after an overnight fast (>10 h). A snack high in fat (muffin) was provided upon initial arrival. Water intake was allowed ad libitum. Upon arrival at the laboratory, participants had to sit down for at least 15 min before an intravenous cannula was inserted into the area of the cubital fossa. Blood sampling was repeated; one sample was obtained at baseline and the others were obtained at 120 and 180 min following ingestion of the capsules. Blood was drawn into lithium–heparin and KEDTA vacutainers for analysis of plasma samples. Plasma was separated (centrifuged at 4 °C on 3500 rpm for 10 min) before being frozen in a −20 °C research refrigerator. Blood pressure and heart rate were measured in triplicate every 10 min throughout the study. Office blood pressures were measured during each visit according to guidelines for blood pressure measurement, using WatchBP Home A (Microlife AG Swiss Corporation, Widnau, Switzerland). Blood pressure was measured in the seated position 3 times during each visit, and the mean value was reported. Urine samples were collected upon arrival each morning and at the end of the protocol. Two urine samples (10 mL) and plasma samples (1.5–2 mL) were used for further analysis. The 24 h food log was reviewed, and a copy was provided to participants who were asked to repeat the exact diet 24 h before the following visit. Subjects were reminded not to perform exercise or to consume alcohol 24 h before the next visit. Subjects returned fasted to the laboratory at least 4 days after the second visit. Adherence to the 24 h diet was confirmed upon arrival in the laboratory. Participants were instructed to identify any deviations in diet from the food log before beginning the previous trial. The protocol was repeated on the third visit, with the participant receiving one of the other two conditions.

### 4.4. Supplementation and Dosing

Each participant received in a randomized and double-blinded order: cannabidiol-matched placebo tablets (labeled as substance ***A***) (12 capsules with 25 mg of active substance each, with total amount of 300 mg of CBD) and DehydraTECH™2.0 CBD (labeled as substance ***B***) (12 capsules with 25 mg of active substance each, with total amount of 300 mg of CBD). Each visit was separated by 14 ± 3 days (range: 11–22). For dose A, participants consumed the capsules 35 ± 16 min after eating the snack (before which they had fasted for 10.3 ± 4.8 h). For dose B, participants consumed the capsules 32 ± 10 min after eating the snack (before which they had fasted for 9.8 ± 4.6 h).

There was no significant difference between these timings between doses (*p* = 0.642 for capsule consumption time; *p* = 0.538 for fasted time; paired *t*-test). It should be noted that: 19 participants had the snack on both visits; two participants did not have a snack on either visit; three subjects only had a snack on one visit (two were dose A, and one was dose B). For both experimental visits, the ingestion of capsules was coordinated to occur at approximately the same time of day. Only three subjects had a between-visit ingestion time >45 min. However, the timing of testing for these three participants still coincided with both either being in the morning or in the afternoon.

DehydraTECH™2.0 CBD delivery technology is a patented capsule formulation that increases the bioabsorption of the active content due to its enhanced lipophilic composition, including a patented process by which long-chain fatty acids, high in oleic acid, are associated through a dehydration process procedure with the CBD. It is believed that this proprietary process assists the human GI system in the uptake of the CBD via bypassing (or reducing) first-pass liver metabolism; therefore, in the short term, it was speculated that this approach allows for higher volumes of CBD to enter the system more rapidly, circumventing first-pass liver metabolism than what is otherwise achieved with generic forms of CBD [19,21,33].

### 4.5. Sample Collection and Storage

Blood samples were collected in lithium heparin Vacutainers. Within 10–15 min of collection, plasma was separated (centrifuged at 4 °C on 3500 rpm for 10 min) and stored at −20 °C. All samples were labeled with the study number for each patient, test session number, date and time. Three plasma samples (1.5–2 mL) and two urine samples (5–10 mL), per patient, were sent directly to the laboratory for analysis.

### 4.6. Standard Solution

Cannabidiol standard solutions (CBD, 1 mg/mL certified reference material; Lipomed AG, Arlesheim, Switzerland) were prepared by dilution with acetonitrile to a final concentration of 10 μg/mL and were used to prepare calibration samples. To establish linearity, a calibration curve was calculated by analyzing drug free plasma and urine samples spiked with CBD at the concentrations of 10, 25, 50, 100, 250 and 500 ng/mL.

### 4.7. CBD Extraction

Proteins in plasma samples (1 mL aliquots) were precipitated with 1.25 mL of ice-cold acetonitrile. After mixing, samples were centrifuged (2600 rpm for 2 min) and supernatant was centrifuged with 1 mL dd H_2_O, as well as the urine sample, which was added to preconditioned solid phase extraction (SPE) columns with CBD specific cartridges (United Chemical Technologies, Styre Screen SSTHC06Z, Bristol, PA, USA; for both, plasma and urine extraction protocol were carried out according to the manufacturer’s instructions). The column was rinsed with 1 mL of dd H_2_O and dried under high vacuum (~20 inches). CBD was eluted with a 3 mL mix of hexane: ethyl acetate: acetic acid (49:49:2, *v*/*v*) and dried under nitrogen. Samples were reconstituted with 150 μL ethyl acetate [19].

### 4.8. GC-MS Analysis

CBD concentration was determined by a gas chromatograph model 8890 GC (Agilent Inc., Santa Clara, CA, USA) coupled to the tandem mass spectrometer model 7000D GC/TQ GC-MS/MS (Agilent Inc., Santa Clara, CA, USA) equipped with an automatic liquid injector model 7693A (Agilent Inc., Santa Clara, CA, USA). A non-polar HP-5MS UI column (dimensions: 30 m long, 0.25 mm inner diameter and 0.25 mm stationary phase layer thickness, Agilent Inc., Santa Clara, CA, USA) was used.

The initial column temperature of 200 °C was held for 1 min, then ramped to 290 °C at 12 °C min^−1^, and then ramped again to 310 °C at 30 °C min^−1^ and held to the total run time of 14.1 min. For the carrier gas, ultrapure-grade helium was used at the flow rate of 1.5 mL min^−1^. The volume of the analyzed sample was 1 μL, and samples were injected using the splitless mode with an injection temperature of 250 °C. GC/MS analysis was performed using single ion monitoring mode (SIM mode) with CBD characteristic ions of 231, 246, 314, 232 and 121 *m*/*z* [40,41].

### 4.9. SNP Genotyping

Blood samples for DNA analysis were collected at regular check-ups and stored in EDTA tubes. DNA was isolated with a commercial genomic DNA isolation kit (High Pure PCR Template Preparation Kit, Version 27, Cat. No. 11796828001, Roche Diagnostics GmbH, Mannheim, Germany) according to the manufacturer’s instructions. Extracted DNA was quantified using a Qubit 4 fluorimeter (Thermo Fischer Scientific, Waltham, MA, USA).

Using the TaqMan^®^ SNP genotyping assay (Thermo Fischer Scientific, Waltham, MA, USA) and an Applied Biosystems 7500 Realtime polymerase chain reaction (RT-PCR) system (Applied Biosystems, Foster City, CA, USA), we genotyped for the following single-nucleotide polymorphisms (SNPs): rs1799853; C_25625805_10; CYP2C9*2, rs1057910; C_27104892_10; CYP2C9*3, rs 4244285; C_25986767_70 CYP2C19*2, rs 4986893; CYP2C19*3, rs 12248560; C_469857_10 CYP2C19*17, and rs2740574; C_1837671_50 *CYP3A4* [21,30,31]. RT-PCR and allelic discrimination analyses were performed according to the manufacturer’s instructions in a 25 mL reaction volume. The temperature program for RT-PCR was 60 °C for 1 min and 95 °C for 10 min, which was followed by 50 cycles of 92 °C for 15 s and 60 °C for 90 s. SNP genotypes were determined using instrument software with the manual allele call option.

### 4.10. Statistical Analysis

The Kolmogorov–Smirnov test was used for normality checking. Due to the non-normal distribution of the data, continuous variables are presented with the median (interquartile range, IQR) and categorical variables are presented with frequencies (percentages). Groups were compared using the non-parametric Mann–Whitney U test, one-way ANOVA test and paired samples *t*-test [42]. Furthermore, multivariate linear regression analysis was performed to assess the association of CYP genotypes with CBD levels in the plasma and urine. The multivariate linear regression model was carried out separately for CBD and DehydraTECH™2.0 CBD doses. CBD levels were logarithmically transformed to achieve normal distribution. In the model, logarithmically transformed CBD levels were dependent variables, while CYP2C9*2, CYP2C9*3, CYP2C19*2, and CYP2C19*17 were independent variables. The model was adjusted for sex. Correlations between changes in the systolic and diastolic blood pressure as well as heart rate with the CBD levels were determined using the Spearman rho correlation coefficient. *p*-values of less than 0.05 were considered statistically significant. Statistical analysis was performed using Statistical Package Software for Social Science, version 28 (SPSS Inc., Chicago, IL, USA).

Using an online calculator, the Hardy–Weinberg equilibrium, χ^2^ and *p* values were calculated [43].

## 5. Conclusions

In conclusion, this research has detected a significant reduction in diastolic blood pressure and MAP in the first 20 min after ingestion of DehydraTECH™2.0 CBD formulation exclusively, while both tested formulations led to a decrease in heart rate. After 180 min of DehydraTECH™2.0 CBD formulation intake, male subjects had higher levels of CBD in their urine compared to female subjects. In the CYP2C9*2*3 enzyme, subjects with the poor metabolizer (PM) phenotype, after consuming the DehydraTECH™2.0 CBD formulation, had a higher plasma concentration of CBD in 180 min.

In future scientific endeavors, it would be optimal to conduct additional trials on a higher number of subjects to measure CBD concentrations and its metabolites after ingesting the novel DehydraTECHTM2.0 CBD formulation in order to better understand the subtle metabolic differences observed thus far.

## Figures and Tables

**Table 1:** Visual analog scale (VAS) summary.

	Dose	BL	90 min	180 min	Dose	Time	Inter
GI	A	0 ± 0	0.8 ± 4.1	1.3 ± 6.1	0.154	0.464	0.579
	B	0 ± 0	0 ± 0	0.1 ± 0.6			
Anxiety	A	8.1 ± 14	3.3 ± 8.6	3.3 ± 8.6	0.523	0.083	0.663
	B	5.5 ± 10.1	3.2 ± 7.8	3.6 ± 7.9			
Sleepiness	A	7.1 ± 14.2	32.9 ± 27.6	29.1 ± 21.9	0.380	<0.001	0.763
	B	3.6 ± 8.5	26.8 ± 30.9	28.6 ± 28.2			

Data presented as mean ± SD. Abbreviation: SD, standard deviation; formulation A, CBD; formulation B, DehydraTECH™2.0 CBD; BL, baseline.

**Table 2:** Descriptive statistics concentration of CBD (A) and DehydraTECH™2.0 CBD (B).

Samples n = 24		Minimum ng/mL	Maximum ng/mL	Median	IQR	Mann–Whitney U Test *p* Value
Plasma at 120 min.	A	5.91	225.22	73.73	99.80	0.853
	B	0.05	715.91	65.79	83.73	
Plasma at 180 min.	A	44.03	807.24	177.48	132.45	0.322
	B	51.22	612.98	134.78	105.76	
Urine at 180 min.	A	8.49	3678.50	545.76	1000.02	0.680
	B	12.16	3750.64	327.35	971.45	

Abbreviation: formulation A, CBD; formulation B, DehydraTECH™2.0 CBD, n, number of subjects exposed; IQR, interquartile range.

**Table 3:** Blood pressure at baseline and 180 min from the start with CBD and DehydraTECH™2.0 CBD formulation.

CBD Formulation	Parameters	Baseline (Mean ± SD)	At 120 min (Mean ± SD)	At 180 min (Mean ± SD)	*p* * (Baseline—120 min)	*p* * (Baseline—180 min)
CBD (n = 24)	Diastolic BP (mmHg)	84.08 ± 8.53	84.58 ± 8.69	85.54 ± 8.78	0.842	0.562
	Systolic BP (mmHg)	136.21 ± 10.54	131.67 ± 10.36	133.5 ± 10.8	0.156	0.403
	Heart rate (bpm)	73.33 ± 11.02	66.54 ± 9.38	66.79 ± 8.41	0.238	0.024
DehydraTECH™2.0 CBD (n = 24)	Diastolic BP (mmHg)	84.38 ± 7.76	84.42 ± 6.58	85.58 ± 8.68	0.984	0.614
	Systolic BP (mmHg)	134.67 ± 9.25	130.17 ± 7.52	132.58 ± 9.82	0.071	0.453
	Heart rate (bpm)	74.33 ± 11.90	68.08 ± 9.34	66.96 ± 9.18	0.048	0.020

* one way ANOVA. Data presented as mean ± SD. Abbreviation: SD, standard deviation; CBD, cannabidiol.

**Table 4:** Genotype and allele frequencies (%) by loci for single-nucleotide polymorphisms (SNPs) of analyzed genes. Data for 24 participants.

Gene	SNP	Genotype	N	Minor Allele	Minor Allele Frequency (MAF) %	Hardy–Weinberg Equilibrium	
						*p* Allele Frequency	χ^2^ *p*-Value
*CYP3A4*	rs2740574	TT	23	C	2.08	0.977	0.011 0.917
		CT	1				
		CC	0				
CYP2C9*2	rs1799853	CC	17	T	16.67	0.833	0.24 0.624
		CT	6				
		TT	1				
CYP2C9*3	rs1057910	AA	19	C	12.50	0.875	1.361 0.243
		AC	4				
		CC	1				
CYP2C19*2	rs4244285	GG	19	A	10.42	0.896	0.325 0.569
		GA	5				
		AA	0				
CYP2C19*3	rs4986893	GG	24	A	0	1.00	0.000 0.569
		GA	0				
		AA	0				
CYP2C19*17	rs12248560	CC	15	T	20.83	0.792	0.003 0.959
		CT	8				
		TT	1				

**Table 5:** Anthropometric summary of participants.

	n	Age (Years)	Height (cm)	Weight (kg)	BMI (kg/m^2^)
Females	12	55 ± 8	167 ± 4	81 ± 9	29.2 ± 3.8
Males	12	52 ± 6	182 ± 6	101 ± 15	30.3 ± 4.2
Total	24	54 ± 7	175 ± 9	91 ± 16	29.7 ± 4

Data presented as mean ± SD. Abbreviation: SD, standard deviation; n, number of subjects exposed (n = 24); BMI, body mass index.

**Table 6:** Participant background summary.

Current smokers (n)	0
Used to smoke (n (%))	6
Years since smoked	20 ± 10; range: 6–33
Pack years when smoking	13 ± 5; range: 5–20
Uses CBD (n (%))	1 ^†^
On medications (n (%))	6
Diazepam (n; dose)	1 (5 mg)
Levothyroxin (n; dose)	3 (25 µg and 100 µg)
Lorazepam (n; dose)	1 (1 mg)
Celecoxib (n; dose)	1 (200 mg)
Acidum salicylicum (n; dose)	1 (100 mg)

Abbreviation: n, number of subjects exposed; CBD, cannabidiol. † Participant had not used CBD products for the 43 days preceding the study. Out of the medications, only Levothyroxin was taken on day of test.

## Data Availability

Data is contained within the article.

## References

[1] Abbotts K.S.S., Ewell T.R., Butterklee H.M., Bomar M.C., Akagi N., Dooley G.P., Bell C. (2022). Cannabidiol and Cannabidiol Metabolites: Pharmacokinetics, Interaction with Food, and Influence on Liver Function. Nutrients.

[2] Soleymanpour M., Saderholm S., Kavuluru R. Therapeutic Claims in Cannabidiol (CBD) Marketing Messages on Twitter. Proceedings of the 2021 IEEE International Conference on Bioinformatics and Biomedicine (BIBM).

[3] Pertwee R.G. (2008). The diverse CB1 and CB2 receptor pharmacology of three plant cannabinoids: Delta9-tetrahydrocannabinol, cannabidiol and delta9-tetrahydrocannabivarin. Br. J. Pharmacol..

[4] Gray R.A., Heal D.J., Maguire D.R., Gerak L.R., Javors M.A., Smith S., France C.P. (2022). Preclinical Assessment of the Abuse Potential of Purified Botanical Cannabidiol: Self-Administration, Drug Discrimination, and Physical Dependence. J. Pharmacol. Exp. Ther..

[5] Jadoon K.A., Tan G.D., O’Sullivan S.E. (2017). A single dose of cannabidiol reduces blood pressure in healthy volunteers in a randomized crossover study. JCI Insight.

[6] Devinsky O., Cross J.H., Laux L., Marsh E., Miller I., Nabbout R., Scheffer I.E., Thiele E.A., Wright S. (2017). Trial of Cannabidiol for Drug-Resistant Seizures in the Dravet Syndrome. N. Engl. J. Med..

[7] Massi P., Solinas M., Cinquina V., Parolaro D. (2013). Cannabidiol as potential anticancer drug. Br. J. Clin. Pharmacol..

[8] Leweke F.M., Piomelli D., Pahlisch F., Muhl D., Gerth C.W., Hoyer C., Klosterkötter J., Hellmich M., Koethe D. (2012). Cannabidiol enhances anandamide signaling and alleviates psychotic symptoms of schizophrenia. Transl. Psychiatry.

[9] McGuire P., Robson P., Cubala W.J., Vasile D., Morrison P.D., Barron R., Taylor A., Wright S. (2018). Cannabidiol (CBD) as an Adjunctive Therapy in Schizophrenia: A Multicenter Randomized Controlled Trial. Am. J. Psychiatry.

[10] Manzanares J., Julian M., Carrascosa A. (2006). Role of the cannabinoid system in pain control and therapeutic implications for the management of acute and chronic pain episodes. Curr. Neuropharmacol..

[11] Devinsky O., Marsh E., Friedman D., Thiele E., Laux L., Sullivan J., Miller I., Flamini R., Wilfong A., Filloux F. (2016). Cannabidiol in patients with treatment-resistant epilepsy: An open-label interventional trial. Lancet Neurol..

[12] Khalsa J.H., Bunt G., Blum K., Maggirwar S.B., Galanter M., Potenza M.N. (2022). Review: Cannabinoids as Medicinals. Curr. Addict. Rep..

[13] Sultan S.R., Millar S.A., England T.J., O’Sullivan S.E. (2017). A Systematic Review and Meta-Analysis of the Haemodynamic Effects of Cannabidiol. Front. Pharmacol..

[14] Rosenberg E.C., Louik J., Conway E., Devinsky O., Friedman D. (2017). Quality of Life in Childhood Epilepsy in pediatric patients enrolled in a prospective, open-label clinical study with cannabidiol. Epilepsia.

[15] Sultan S.R., O’Sullivan S.E., England T.J. (2020). The effects of acute and sustained cannabidiol dosing for seven days on the haemodynamics in healthy men: A randomised controlled trial. Br. J. Clin. Pharmacol..

[16] Taylor L., Gidal B., Blakey G., Tayo B., Morrison G. (2018). A Phase I, Randomized, Double-Blind, Placebo-Controlled, Single Ascending Dose, Multiple Dose, and Food Effect Trial of the Safety, Tolerability and Pharmacokinetics of Highly Purified Cannabidiol in Healthy Subjects. CNS Drugs.

[17] Watkins P.B., Church R.J., Li J., Knappertz V. (2021). Cannabidiol and Abnormal Liver Chemistries in Healthy Adults: Results of a Phase I Clinical Trial. Clin. Pharmacol. Ther..

[18] Millar S.A., Maguire R.F., Yates A.S., O’Sullivan S.E. (2020). Towards Better Delivery of Cannabidiol (CBD). Pharmaceuticals.

[19] Patrician A., Versic-Bratincevic M., Mijacika T., Banic I., Marendic M., Sutlović D., Dujić Ž., Ainslie P.N. (2019). Examination of a New Delivery Approach for Oral Cannabidiol in Healthy Subjects: A Randomized, Double-Blinded, Placebo-Controlled Pharmacokinetics Study. Adv. Ther..

[20] Kumric M., Dujic G., Vrdoljak J., Svagusa K., Kurir T.T., Supe-Domic D., Dujic Z., Bozic J. (2023). CBD supplementation reduces arterial blood pressure via modulation of the sympatho-chromaffin system: A substudy from the HYPER-H21-4 trial. Biomed. Pharm..

[21] Kumric M., Bozic J., Dujic G., Vrdoljak J., Dujic Z. (2022). Chronic Effects of Effective Oral Cannabidiol Delivery on 24-h Ambulatory Blood Pressure and Vascular Outcomes in Treated and Untreated Hypertension (HYPER-H21-4): Study Protocol for a Randomized, Placebo-Controlled, and Crossover Study. J. Pers. Med..

[22] Jiang R., Yamaori S., Takeda S., Yamamoto I., Watanabe K. (2011). Identification of cytochrome P450 enzymes responsible for metabolism of cannabidiol by human liver microsomes. Life Sci..

[23] Bachtiar M., Lee C.G.L. (2013). Genetics of Population Differences in Drug Response. Curr. Genet. Med. Rep..

[24] Sukprasong R., Chuwongwattana S., Koomdee N., Jantararoungtong T., Prommas S., Jinda P., Rachanakul J., Nuntharadthanaphong N., Jongjitsook N., Puangpetch A. (2021). Allele frequencies of single nucleotide polymorphisms of clinically important drug-metabolizing enzymes CYP2C9, CYP2C19, and CYP3A4 in a Thai population. Sci. Rep..

[25] Pratt V.M., Cavallari L.H., Del Tredici A.L., Hachad H., Ji Y., Moyer A.M., Scott S.A., Whirl-Carrillo M., Weck K.E. (2019). Recommendations for Clinical CYP2C9 Genotyping Allele Selection: A Joint Recommendation of the Association for Molecular Pathology and College of American Pathologists. J. Mol. Diagn. JMD.

[26] Ionova Y., Ashenhurst J., Zhan J., Nhan H., Kosinski C., Tamraz B., Chubb A. (2020). CYP2C19 Allele Frequencies in Over 2.2 Million Direct-to-Consumer Genetics Research Participants and the Potential Implication for Prescriptions in a Large Health System. Clin. Transl. Sci..

[27] Zanger U.M., Schwab M. (2013). Cytochrome P450 enzymes in drug metabolism: Regulation of gene expression, enzyme activities, and impact of genetic variation. Pharmacol. Ther..

[28] Nasrin S., Watson C.J.W., Perez-Paramo Y.X., Lazarus P. (2021). Cannabinoid Metabolites as Inhibitors of Major Hepatic CYP450 Enzymes, with Implications for Cannabis-Drug Interactions. Drug Metab. Dispos. Biol. Fate Chem..

[29] PharmGKB. https://www.pharmgkb.org/vip/PA166169770.

[30] Bozina N., Granić P., Lalić Z., Tramisak I., Lovrić M., Stavljenić-Rukavina A. (2003). Genetic polymorphisms of cytochromes P450: CYP2C9, CYP2C19, and CYP2D6 in Croatian population. Croat. Med. J..

[31] Bonomo Y., Norman A., Collins L., O’Neill H., Galettis P., Trinca J., Strauss N., Martin J., Castle D. (2022). Pharmacokinetics, Safety, and Tolerability of a Medicinal Cannabis Formulation in Patients with Chronic Non-cancer Pain on Long-Term High Dose Opioid Analgesia: A Pilot Study. Pain Ther..

[32] Millar S.A., Stone N.L., Yates A.S., O’Sullivan S.E. (2018). A Systematic Review on the Pharmacokinetics of Cannabidiol in Humans. Front. Pharmacol..

[33] Bioscience L. DehydraTECH. https://lexariabioscience.com/.

[34] Skadrić I., Stojković O. (2020). Defining screening panel of functional variants of CYP1A1, CYP2C9, CYP2C19, CYP2D6, and CYP3A4 genes in Serbian population. Int. J. Leg. Med..

[35] Beers J.L., Fu D., Jackson K.D. (2021). Cytochrome P450-Catalyzed Metabolism of Cannabidiol to the Active Metabolite 7-Hydroxy-Cannabidiol. Drug Metab. Dispos. Biol. Fate Chem..

[36] PharmGKB. https://www.pharmgkb.org/labelAnnotation/PA166104784.

[37] PharmGKB. https://www.pharmgkb.org/labelAnnotation/PA166127647.

[38] Takahashi N., Inui N., Morita H., Takeuchi K., Uchida S., Watanabe H., Nakamura H. (2010). Effect of thyroid hormone on the activity of CYP3A enzyme in humans. J. Clin. Pharmacol..

[39] Flack J.M., Adekola B. (2020). Blood pressure and the new ACC/AHA hypertension guidelines. Trends Cardiovasc. Med..

[40] Sutlović D., Sutlović D. (2011). Osnove Forenzične Toksikologije, Potvrdne Metode Analize.

[41] Sutlović D. (2011). Osnove Forenzične Toksikologije, Potvrdna Analitička Metoda, Plinska Kromatografija-Spektrometrija Masa.

[42] One Way Anova Calculator. https://goodcalculators.com/one-way-anova-calculator/.

[43] Hardy-Weinberg Equilibrium. http://www.dr-petrek.eu/documents/HWE.xls.

